# Identification of Pathologic and Prognostic Genes in Prostate Cancer Based on Database Mining

**DOI:** 10.3389/fgene.2022.854531

**Published:** 2022-03-11

**Authors:** Kun Liu, Yijun Chen, Pengmian Feng, Yucheng Wang, Mengdi Sun, Tao Song, Jun Tan, Chunyang Li, Songpo Liu, Qinghong Kong, Jidong Zhang

**Affiliations:** ^1^ Department of Immunology, Zunyi Medical University, Zunyi, China; ^2^ School of Basic Medical Sciences, Chengdu University of Traditional Chinese Medicine, Chengdu, China; ^3^ Department of Histology and Embryology, Zunyi Medical University, Zunyi, China; ^4^ Guizhou Provincial College-based Key Lab for Tumor Prevention and Treatment with Distinctive Medicines, Zunyi Medical University, Zunyi, China; ^5^ Special Key Laboratory of Gene Detection and Therapy of Guizhou Province, Zunyi Medical University, Zunyi, China

**Keywords:** prostate cancer, prognosis, biomarker, TCGA, differentially expressed genes

## Abstract

**Background:** Prostate cancer (PCa) is an epithelial malignant tumor that occurs in the urinary system with high incidence and is the second most common cancer among men in the world. Thus, it is important to screen out potential key biomarkers for the pathogenesis and prognosis of PCa. The present study aimed to identify potential biomarkers to reveal the underlying molecular mechanisms.

**Methods:** Differentially expressed genes (DEGs) between PCa tissues and matched normal tissues from The Cancer Genome Atlas Prostate Adenocarcinoma (TCGA-PRAD) dataset were screened out by R software. Weighted gene co-expression network analysis was performed primarily to identify statistically significant genes for clinical manifestations. Protein–protein interaction (PPI) network analysis and network screening were performed based on the STRING database in conjunction with Cytoscape software. Hub genes were then screened out by Cytoscape in conjunction with stepwise algorithm and multivariate Cox regression analysis to construct a risk model. Gene expression in different clinical manifestations and survival analysis correlated with the expression of hub genes were performed. Moreover, the protein expression of hub genes was validated by the Human Protein Atlas database.

**Results:** A total of 1,621 DEGs (870 downregulated genes and 751 upregulated genes) were identified from the TCGA-PRAD dataset. Eight prognostic genes [*BUB1*, *KIF2C*, *CCNA2*, *CDC20*, *CCNB2*, *PBK*, *RRM2*, and *CDC45*] and four hub genes (*BUB1*, *KIF2C*, *CDC20*, and *PBK*) potentially correlated with the pathogenesis of PCa were identified. A prognostic model with good predictive power for survival was constructed and was validated by the dataset in GSE21032. The survival analysis demonstrated that the expression of *RRM2* was statistically significant to the prognosis of PCa, indicating that *RRM2* may potentially play an important role in the PCa progression.

**Conclusion:** The present study implied that *RRM2* was associated with prognosis and could be used as a potential therapeutic target for PCa clinical treatment.

## Introduction

Prostate cancer (PCa), one of the most common neoplasms worldwide, is ranked fourth among all cancer types with an incidence of 7.1% (Bray et al., 2018) and has been reported to be the second leading cause of cancer death among men (Siegel et al., 2018). With the increasing diagnosis of PCa in China and the aging of the population, several urologists have performed abundant research on PCa and prostatic diseases (Ye and Zhu, 2019; Li J. et al., 2020).

Nowadays, the detection of serum prostate-specific antigen (PSA) has been considered a common method to screen prostate cancer in the clinic. Nevertheless, due to its low specificity and significant limitations, it is difficult to make an early diagnosis with PSA. Furthermore, it is painful for patients to undergo traditional prostate biopsy, which is regarded as the golden standard of PCa. Moreover, the traditional treatment methods for prostate cancer were endocrine therapy, radiotherapy, surgery, and chemotherapy (Sia et al., 2008; Moore et al., 2009). However, the prognosis differs among various patients, and it is difficult to predict the prognosis simply by clinical information. Therefore, novel biomarkers are needed for the pathogenesis and prognosis of prostate cancer.

Weighted gene co-expression network analysis (WGCNA) has been widely used to identify co-expressed modules and hub genes (Langfelder and Horvath, 2008). In this study, we aimed to screen potential key genes correlated with the pathogenesis and prognostic biomarkers through bioinformatics analysis. First, differentially expressed genes (DEGs) in TCGA-PRAD RNA-seq data were screened. Then, the DEGs of key modules clustered by WGCNA were selected for further analysis. Gene Ontology (GO) and Kyoto Encyclopedia of Genes and Genomes (KEGG) pathway analysis for the overlapping differentially expressed genes were annotated by R software PPI network and Cytoscape. Finally, a risk model and survival analysis were performed based on the candidate key genes.

## Materials and Methods

### Data Acquisition and Processing

The TCGA database is a public funded project that aims to catalog and discover major cancer-causing genomic alterations to create a comprehensive “atlas” of cancer genomic profiles (Tomczak et al., 2015). The TCGA-PRAD dataset was screened out from UCSC Xena (https://xena.ucsc.edu/). The raw data were preprocessed with the following criteria: 1) samples without related clinical data (age, Gleason grade, N stage, and T stage) or survival data were removed; 2) genes with missing expression values in more than half of the samples were excluded; 3) genes were excluded if the FPKM value (fragments per kilobase million) was 0 in more than half of the samples; and 4) genes were screened out if the FPKM value was more than 1 in more than 10% of the samples. GSE21032 containing the normalized log_2_ mRNA expression data and clinical characteristics (131 primary prostate cancer samples and 50 normal samples) was downloaded from c-BioPortal (http://cbioportal.org). R software (version 4.0.2) and SPSS software (version 23.0) were used in the subsequent analysis.

### Identification of Differentially Expressed Genes

DEGs between tumor samples and matched normal samples were screened out using Wilcoxon rank sum tests in R software (version 4.0.2) with *p* < 0.05 and log_2_|FC| > 1. The volcano plot of DEGs was plotted by the R package “ggplot2” (version 3.3.5).

### Construction of Weighted Gene Co-Expression Network Analysis for Key Module Mining

A weighted co-expression network of DEGs screened out from the dataset was constructed to explore the biological function by the R package “WGCNA” (version 1.70–3). The best soft-thresholding was determined automatically by the R package “WGCNA.” Interaction of different co-expression modules was assessed, and the heatmap was constructed. Based on the heatmap, genes in the key modules were screened out for further analysis.

### Pathway Enrichment Analysis of Differentially Expressed Genes

Gene ontology (GO) enrichment analysis plays an important role in describing the biological processes (BPs), cellular components (CCs), and molecular functions (MFs) correlated with DEGs (Chen et al., 2017). KEGG pathway analysis plays an important role in describing biological pathways correlated with DEGs (Kanehisa and Goto, 2000; Chen et al., 2017). Our analysis was performed based on the DEGs screened out from key modules by the R package “clusterProfiler” (version 3.16.1), *p* < 0.05 and q < 0.05 (Yu et al., 2012).

### Construction of Protein–Protein Interaction Network and Module Mining

The STRING database (http://string-db.org, version 11.5) was widely used for protein–protein interaction (PPI) network construction (Szklarczyk et al., 2021). Interactions among the DEGs in the key modules were calculated based on the STRING database. The interaction with a confidence >0.7 was considered a significant interaction and retained. To identify the hub regulatory genes, the PPI network data were imported into Cytoscape software for further analysis. We found an important cluster module based on MCODE (version 2.0.0) plugin in Cytoscape. Parameter settings were as follows: degree cutoff = 2, node score cutoff = 0.2, K-core = 2, and depth cutoff = 100.

### Pathway Enrichment Analysis of Differentially Expressed Genes in the Key Module and Network Screening

Using R software, GO enrichment analysis and KEGG pathway analysis were performed based on all the genes in the key module. Then, we screened the functionally grouped gene ontology and pathway annotation networks by ClueGO (version 2.5.8) and CluePedia (version 1.5.8) in Cytoscape (Bindea et al., 2009; Bindea et al., 2013). Parameter was limited to simplify the figure and only illustrate the key pathway.

### Hub Gene Mining and Construction of a Prognostic Risk Model

According to the counts of each gene calculated by MCODE, the top 25 candidate hub genes in the key module were screened out by cytoHubba (version 0.1) (Chin et al., 2014). The Cox proportional hazard regression model has achieved widespread use in the analysis of time-to-event data with censoring and covariates (Fisher and Lin, 1999). In our study, the key genes associated with overall survival (OS) were screened out from the top 25 candidate hub genes by Cox proportional hazard regression analysis based on the TCGA dataset. The hazard ratio (HR), 95% confidence interval (CI), Globe *p* value (log rank), and concodence index were calculated to identify potential oncogenes and antioncogenes *via* R function “coxph.” The risk score of each sample was predicted by R software, and the samples were divided into high-risk and low-risk groups by the score cutoff. GSE21032 was used as a test set for validating the risk model constructed based on the TCGA dataset. The forest plot and Kaplan–Meier (KM) survival curve were plotted by the R packages “survival” (version 3.2-11) and “survminer” (version 0.4.9). The risk distribution was plotted by the R package “pROC” (version 1.17.0.1).

### Analysis of Hub Gene Expression and Validation

After construction of the prognostic risk model based on the TCGA dataset and validation based on GSE21032, the expression of candidate genes eventually screened out in different hierarchies of clinical information was screened by the R packages “ggplot2” and “pheatmap” (version 1.0.12), using Wilcoxon rank sum tests in R software. Protein expression correlated with eight hub genes was validated in the Human Protein Atlas (HPA) database (https://www.proteinatlas.org) (Uhlen et al., 2015; Uhlen et al., 2017).

## Results

### Differentially Expressed Gene Identification and Co-Expression Network Construction

**FIGURE 1:**
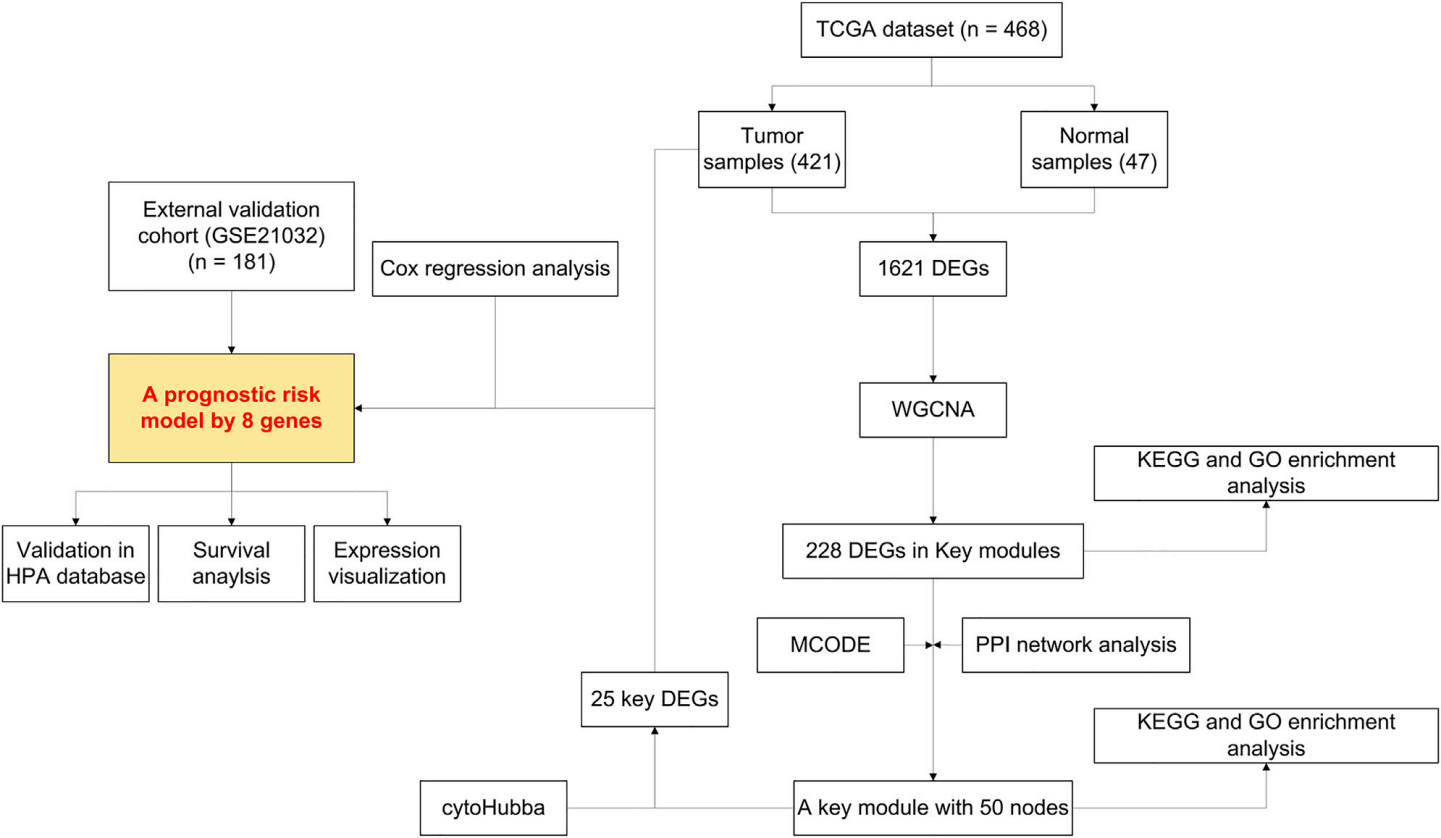
Flowchart presenting the process of establishing the gene signature and prognostic nomogram of prostate cancer in this study.

**FIGURE 2:**
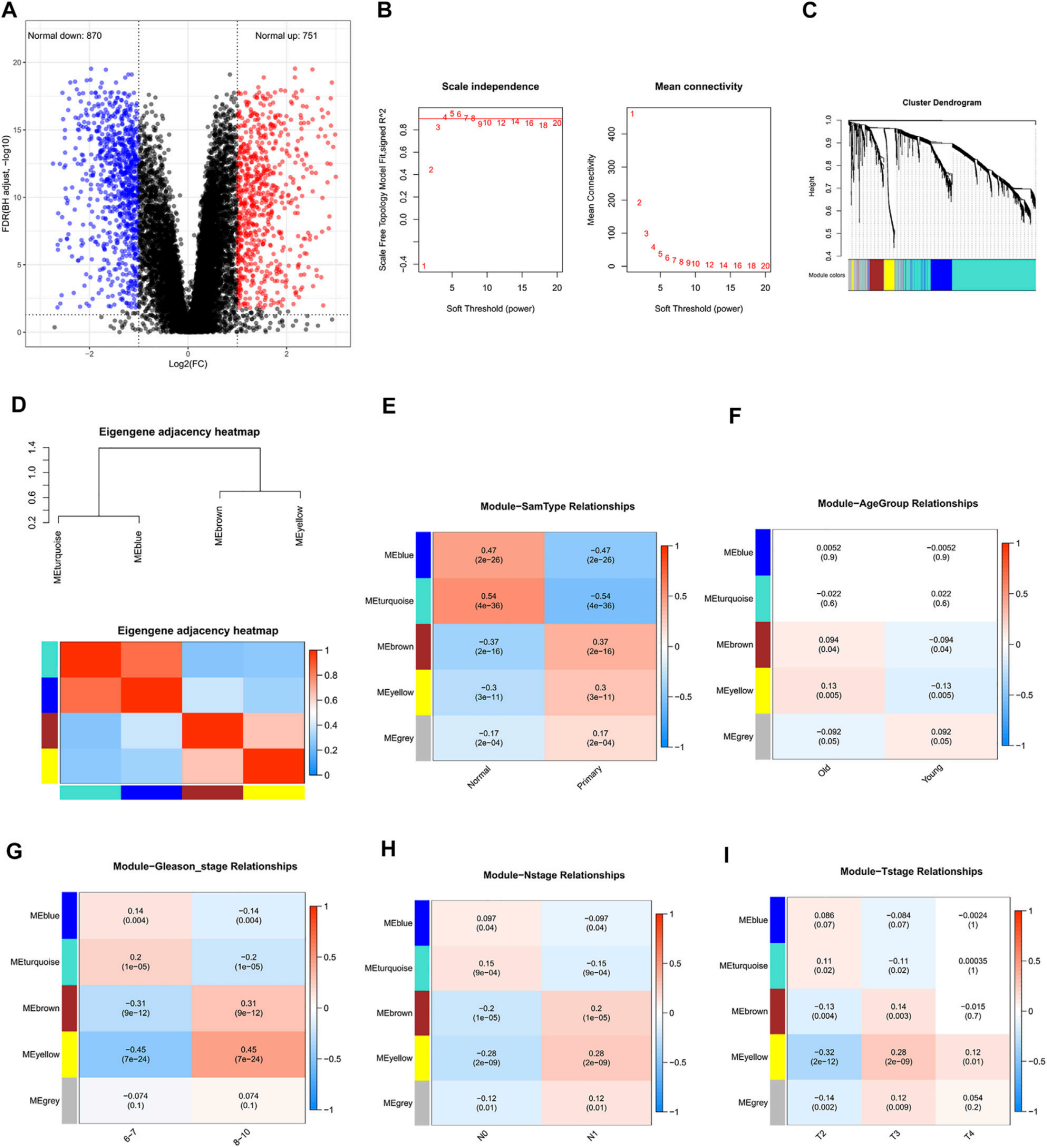
Screening for differentially expressed genes in prostate cancer (PCa) and weighted co-expression network analysis (WGCNA) based on differentially expressed genes. **(A)** Volcano plot of DEGs in TCGA-PRAD cohort. The blue dot represents the downregulated gene, while the red dot represents the upregulated gene. **(B)** Determining soft-thresholding power in WGCNA: the scale-free fit index and the mean connectivity for various soft-thresholding powers. **(C)** Clustering dendrogram and color display of co-expression network modules in the samples. **(D)** The eigengene adjacency heatmap was constructed. **(E)** Heatmap of the correlation between module eigengenes and the sample type of prostate cancer. **(F)** Heatmap of the correlation between module eigengenes and the age group (cutoff: 62) of prostate cancer. **(G)** Heatmap of the correlation between module eigengenes and the Gleason stage of prostate cancer. **(H)** Heatmap of the correlation between module eigengenes and the N stage of prostate cancer. **(I)** Heatmap of the correlation between module eigengenes and the T stage of prostate cancer.

Our study was conducted according to the flow chart shown in Figure 1. In total, 468 samples (47 normal samples and 421 tumor samples) from the GSE21032 dataset were eventually selected and analyzed. All clinical information were arranged in the chart and also shown in Table 1. A total of 1,621 DEGs (870 downregulated and 751 upregulated genes) under the threshold *p* < 0.05 and log_2_|FC| > 1 were screened from the GSE21032 dataset and shown in a volcano plot (Figure 2A, Supplementary Table S1). The obtained 1,621 DEGs were further analyzed and screened with WGCNA to construct a co-expression network. Since the scale-free topology fit index was 0.9 (Figure 2B left) and the lower the mean connectivity number, the better (Figure 2B right), the soft-thresholding (power) = 4 was selected. The best soft-clustered genes were confirmed using a topological overlap matrix (TOM)-based dissimilarity measure by using the dynamic tree cut algorithm, and five gene modules were obtained (Figure 2C). To correlate the resulting modules with the clinic traits, the first principal components of the module matrix were calculated and defined as eigengenes (Langfelder and Horvath, 2008). As shown in Figure 2D, the network heatmap was plotted to indicate the relationship between each module.

**TABLE 1:** TCGA-PRAD patients’ characters.

Clinical character		Total (*n* = 468)	%
Sample type	Normal	47	10.0
	Primary	421	90.0
Age group	≥62	243	51.9
	<62	225	48.1
Gleason stage	6–7	271	57.9
	8–10	197	42.1
N stage	N0	389	83.1
	N1	79	16.9
T stage	T2	170	36.3
	T3	286	61.1
	T4	12	2.6

### Clinically Significant Module Selection

After relating modules to traits, it was found that the genes in MEturquoise and MEblue exhibited higher correlations with sample types than other modules (Figure 2E, Supplementary Tables S2, S3 **)**, indicating that they were significantly (*p* < 0.05) associated with the occurrence of PCa and could be considered as cancer-suppressing genes. Therefore, the genes in MEyellow and MEbrown were selected as the clinically significant modules for further analysis to find hub genes related with PCa, which exhibited high correlations with the sample types, Gleason stage, N stage, and T stage (Figures 2E,2G–I, Supplementary Tables S4, S5), revealing that they were correlated with the occurrence of PCa and the progression of PCa. Since the Pearson correlation coefficients between the two modules (MEyellow and MEbrown) and age were relatively low (0.13 and 0.094, respectively, Figure 2F), genes in these modules might not play important roles in aging.

### Functional Enrichment Annotation and Protein–Protein Interaction Network Construction

**FIGURE 3:**
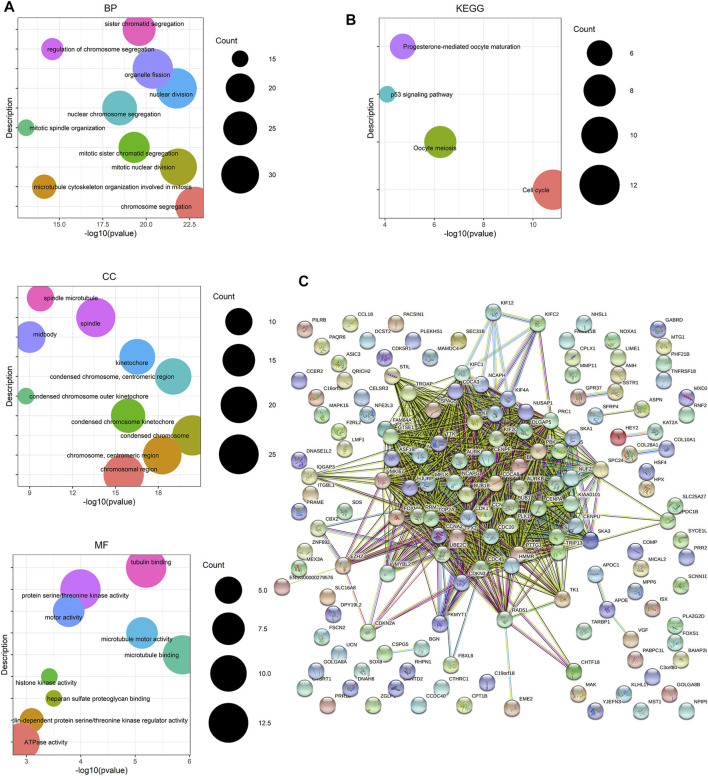
Enrichment analysis and protein–protein interaction (PPI) network based on genes from the yellow and brown module from WGCNA. **(A)** GO term enrichment analysis of DEGs obtained from two modules. The main GO terms (*p* value< 0.05) are shown for the biological process (BP), cellular component (CC), and molecular function (MF). For BP and CC, only results with the top 10 lowest *p* value are shown. **(B)** KEGG pathway enrichment analysis of DEGs obtained from two modules. **(C)** PPI network of the overlapping DEGs constructed based on the STRING online database.

To uncover the hub genes from the candidate genes obtained from MEyellow and MEbrown, functional enrichment analysis and protein–protein interaction (PPI) network construction were performed. The results of GO term enrichment analysis and KEGG pathway enrichment analysis based on DEGs in the two key modules are shown in Figures 3A,B. Notably, GO term enrichment analysis demonstrated that the DEGs were mainly enriched in chromosome segregation, mitotic nuclear division, nuclear division, and so forth. From KEGG pathway enrichment analysis, the DEGs were mainly associated with cell cycle, oocyte meiosis, progesterone-mediated oocyte maturation, and the p53 signaling pathway. The PPI network of DEGs was built by using STRING, which includes 159 nodes and 1,407 edges (Figure 3C). The aforementioned results revealed that the abundant signal pathways in which the DEGs were enriched may play pivotal roles in the pathological processes of PCa.

### Network Visualization of Differentially Expressed Genes in Key Pathways

**FIGURE 4:**
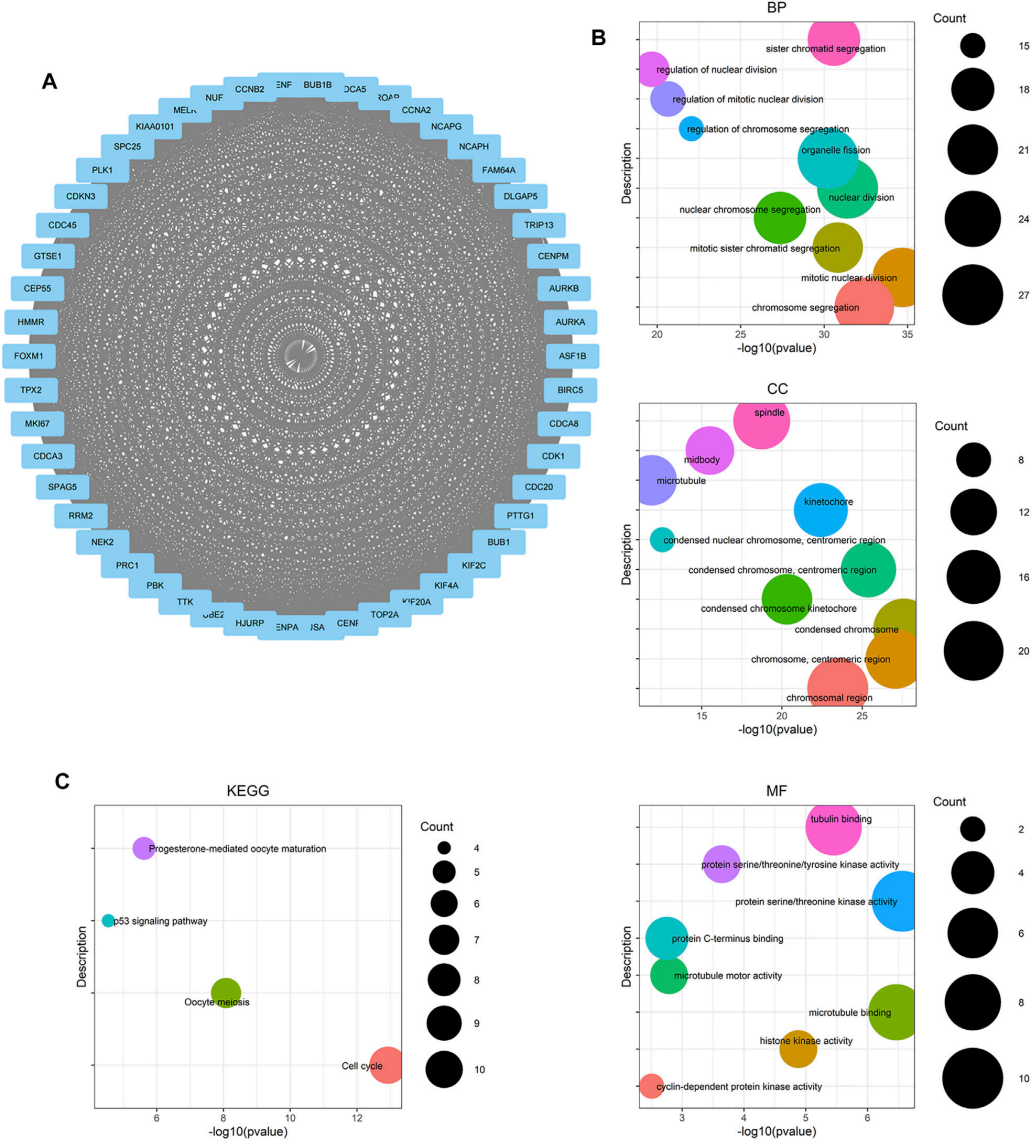
Module analysis based on DEGs from two selected WGCNA modules. **(A)** Molecular Complex Detection (MCODE) plugin in Cytoscape was used to detect cluster modules. One module was screened by using MCODE. The module score is 46.122, with 50 nodes and 1,130 edges. **(B)** GO term enrichment analysis of DEGs obtained from the module. The main GO terms (*p* value< 0.05) are shown for the biological process (BP), cellular component (CC), and molecular function (MF). For BP and CC, only results with the top 10 lowest *p* value are shown. **(C)** KEGG pathway enrichment analysis of DEGs obtained from the module.

**FIGURE 5:**
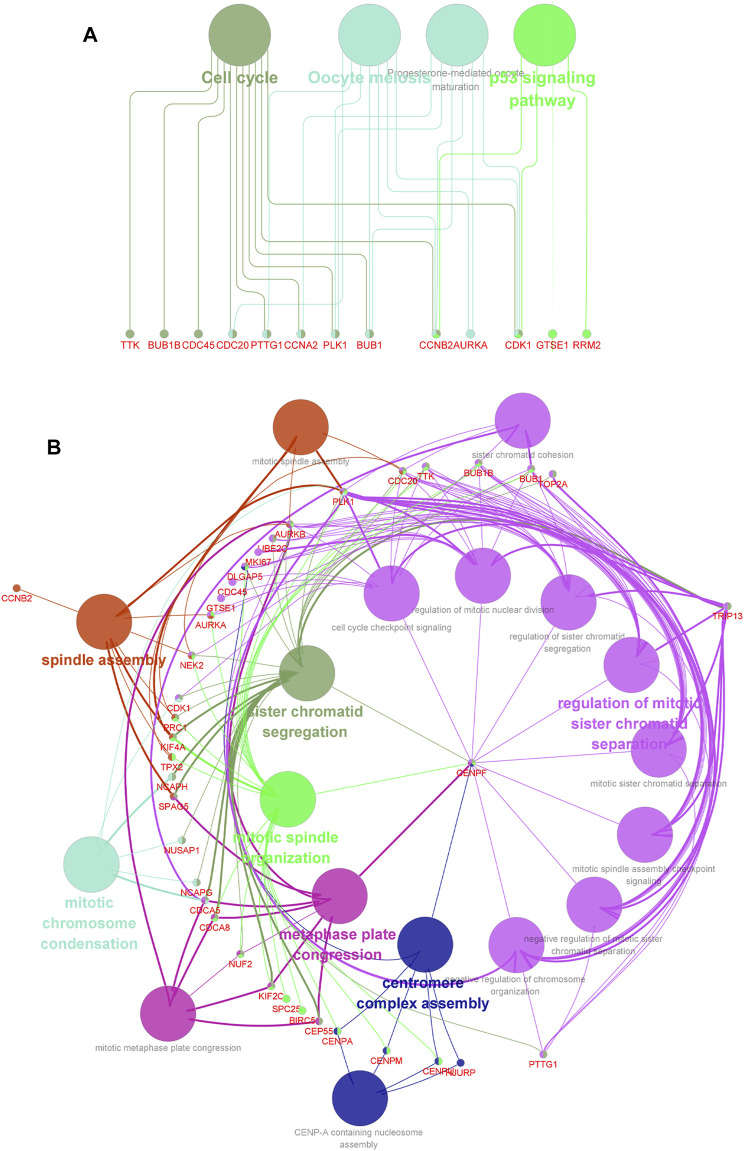
Network relationship screened by using the ClueGO (v2.5.8) and CluePedia plugins (v1.5.8) in Cytoscape, based on DEGs obtained from the module picked out by MCODE. **(A)** Network relationship of GO term enrichment analysis. **(B)** Network relationship of KEGG pathway enrichment analysis. Pathways with the same color indicate that they have similar functions.

To find genes more significantly related with PCa and key relationship network, protein interaction data analyzed by STRING were imported into Cytoscape for further analysis. We found a key module containing 50 nodes and 1,130 edges, which was considered to be significantly associated with PCa and valuable for further analysis (Figure 4A). The gene names and scores of nodes are shown in Supplementary Table S6. To explore the functions and relationships of the potential hub genes, GO term enrichment analysis and KEGG pathway enrichment analysis based on DEGs in the key module were performed (Figures 4B,C). For GO term enrichment analysis, we found that the DEGs were associated with mitotic nuclear division, chromosome segregation, nuclear division, and so forth. In KEGG pathway enrichment analysis, DEGs were enriched in cell cycle, oocyte meiosis, progesterone-mediated oocyte maturation, and the p53 signaling pathway. GlueGO and GluePedia were used in Cytoscape to visualize the enrichment results and found certain genes related to multiple pathways, which may be potential genes playing an important role in regulating the function of the module (Figures 5A,B).

### Identification of Hub Genes and a Risk Model Predicting the Prognosis of PCa

**FIGURE 6:**
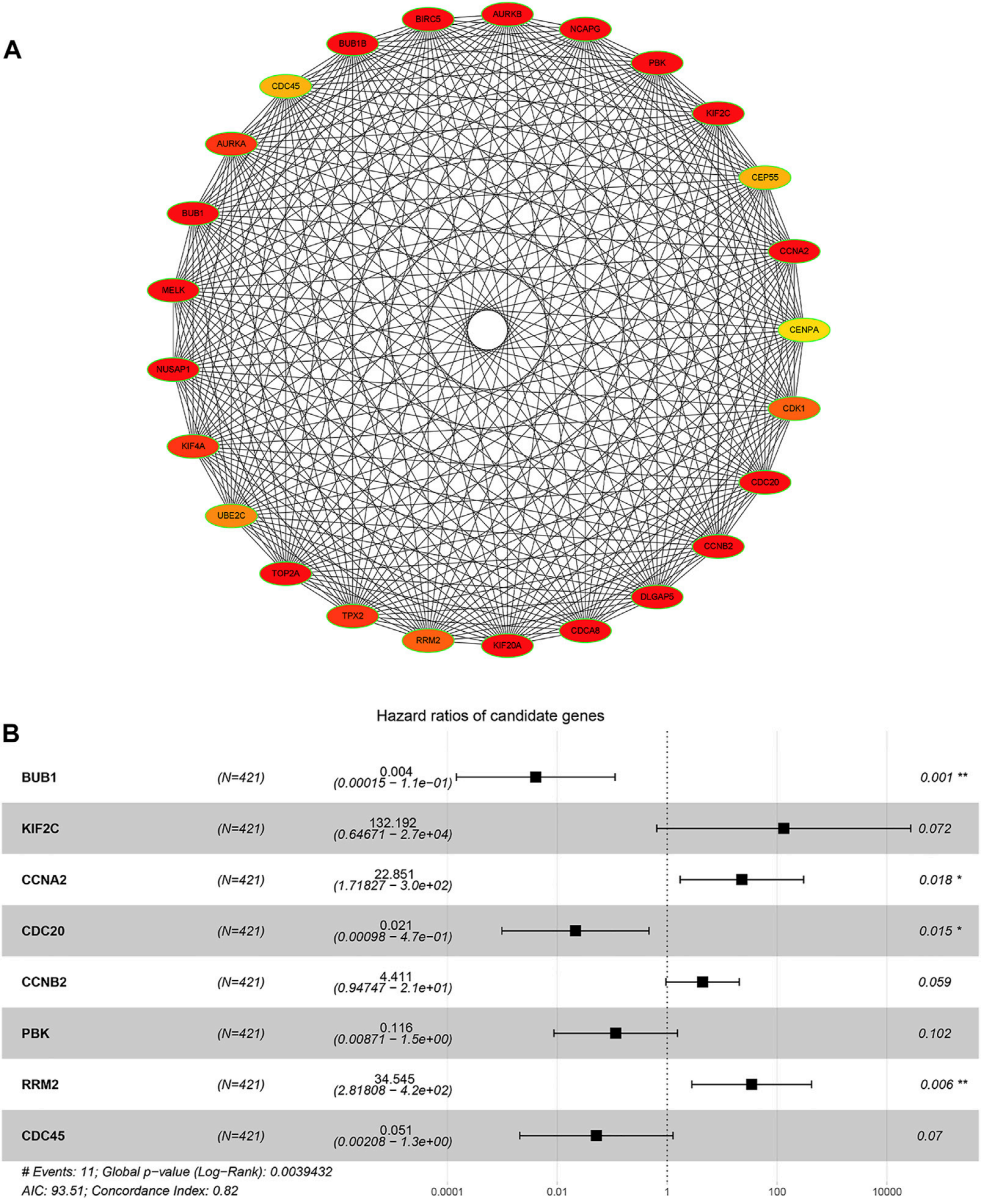
Hub gene identification based on DEGs from the module picked out by MCODE. **(A)** Cytohubba plugin in Cytoscape was used to determine the top 25 hub genes in protein–protein interaction. **(B)** Forest plot of risk factors affecting the survival in PCa patients. Hazard ratio (95% CI) and *p* value of each hub genes are shown.

Twenty-five DEGs with the highest scores in the key module were screened out using cytoHubba plugin in Cytoscape (Figure 6A, Supplementary Table S7). Subsequently, to construct a Cox proportional hazard regression model, eight genes (*BUB1*, *KIF2C*, *CCNA2*, *CDC20*, *CCNB2*, *PBK*, *RRM2*, and *CDC45*) were eventually screened out by the using stepwise algorithm in the R package “stats.” HR and 95% CI of each gene are shown in Table 2. The forest plot was utilized to visualize the multivariate Cox proportional hazard regression model constructed based on the candidate hub genes. As shown in Figure 6B, the concordance (C-index value) was 0.821 (se = 0.062) and the *p* value of the score (log rank) test was 0.004 (Global *p* value). We found that the hazard ratio (HR) value of *BUB1*, *CCNA2*, *CDC20*, and *RRM2* had statistical significance (*p* < 0.05). *BUB1*, *CDC20*, *PBK*, and *CDC45* were potential risk genes with HR < 1, while *KIF2C*, *CCNA2*, *CCNB2*, and *RRM2* are potential protective genes with HR > 1.

**TABLE 2:** Results of multivariate Cox proportional hazard regression analysis.

Gene symbol	Coefficient	Hazard ratio (95% CI)	se (coef)	z	*p* value
*BUB1*	−5.509	4.049e-03 (0.0001–1.127e-01)	1.697	−3.246	**0.001**
*KIF2C*	4.884	1.322e + 02 (0.647–2.702e + 04)	2.714	1.799	0.072
*CCNA2*	3.129	2.285e + 01 (1.718–3.039e + 02)	1.320	2.370	**0.018**
*CDC20*	−3.842	2.144e-02 (0.001–4.669e-01)	1.572	−2.445	**0.015**
*CCNB2*	1.484	4.411e + 00 (0.947–2.053e + 01)	0.785	1.891	0.059
*PBK*	−2.156	1.158e-01 (0.009–1.539e + 00)	1.320	−1.633	0.102
*RRM2*	3.542	3.455e + 01 (2.818–4.235e + 02)	1.279	2.770	**0.006**
*CDC45*	−2.967	5.145e-02 (0.002–1.275e + 00)	1.638	−1.812	0.070

Note: p < 0.05 written with bold number represents the gene may be potentially correlated with the prognosis of prostate cancer.

**FIGURE 7:**
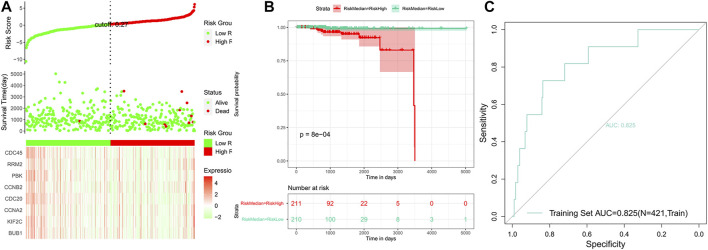
Analysis of the risk model in the train set. **(A)** Distribution of risk scores, survival status, and the eight genes’ expression heat map. **(B)** Kaplan–Meier (KM) curves of the OS in the train set. **(C)** ROC curves and area under the curve (AUC = 0.825) of the risk model.

**FIGURE 8:**
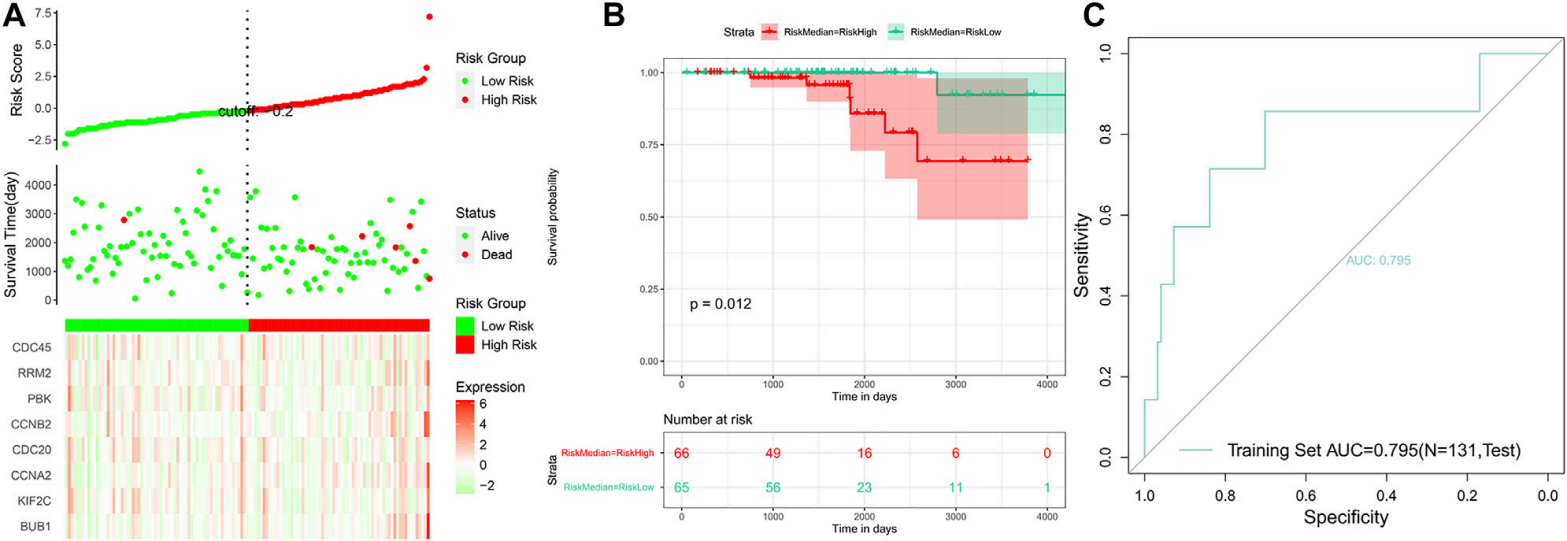
Analysis of the risk model in the test set. **(A)** Distribution of risk scores, survival status, and the eight genes’ expression heatmap. **(B)** Kaplan–Meier (KM) curves of the OS in the test set. **(C)** ROC curves and area under the curve (AUC = 0.795) of the risk model.

Based on the risk scores predicted for each sample in the train set, a total of 421 patients were divided into high-risk and low-risk groups by the median cutoff of risk score 0.27. Distribution of risk scores, survival status, and the candidate hub gene expression heatmap based on the TCGA dataset were plotted (Figure 7A). We compared the OS of the two groups using the Kaplan–Meier (KM) curve with the risk table (*p* < 0.05) (Figure 7B). The prognosis prediction efficiency was analyzed by the ROC curve. The model had a relatively high value of area under the curve (AUC) with a value of 0.825 (AUC >0.8, Figure 7C). The robustness of the risk model was validated on the GEO dataset GSE21032. Distribution of risk scores, survival status, and the eight genes’ expression heatmap in the GEO dataset are shown in Figure 8A. The KM curve with the risk table (*p* < 0.05) and the ROC curve (AUC >0.7) are shown in Figures 8B,C. The results in the train set and test set indicated that it may be possible to predict the prognosis of prostate cancer patients based on the risk score in our model. The eight selected candidate hub genes could be considered the prognostic biomarkers for PCa.

### Expression of the Eight Hub Genes Significantly Differed in Clinical Manifestations

**FIGURE 9:**
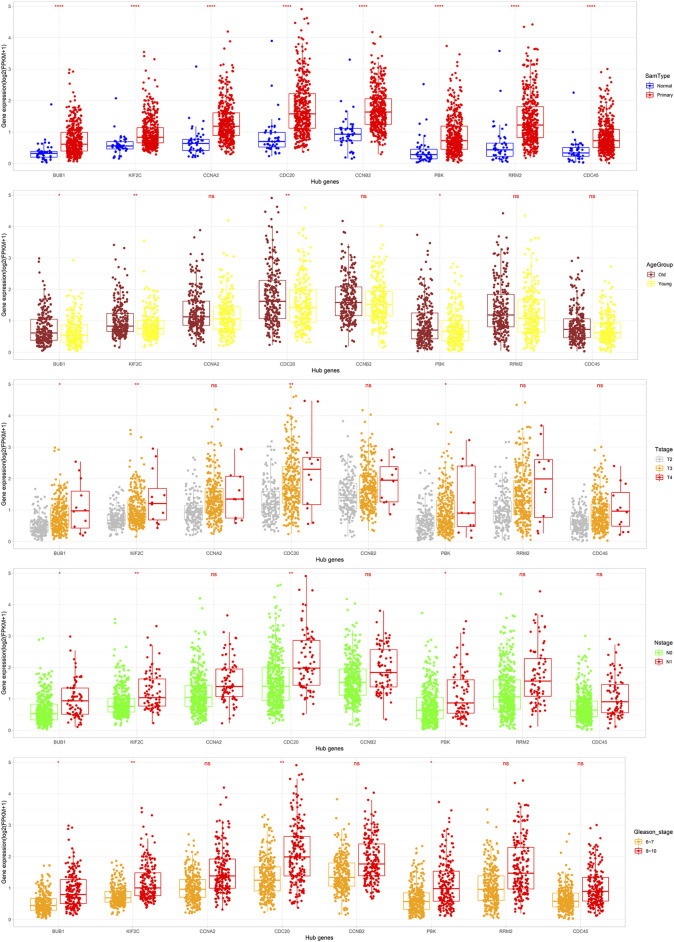
Expression of the eight hub DEGs in different types of tissues, age groups (cutoff = 62), T stages, N stages, and Gleason stages in the TCGA-PRAD dataset. Expression values of the ten hub DEGs are log_2_-transformed (*: *p < 0.05*; **: *p < 0.01*; ***: *p < 0.001*; ****: *p < 0.0001*; and ns: *p > 0.05*).

**FIGURE 10:**
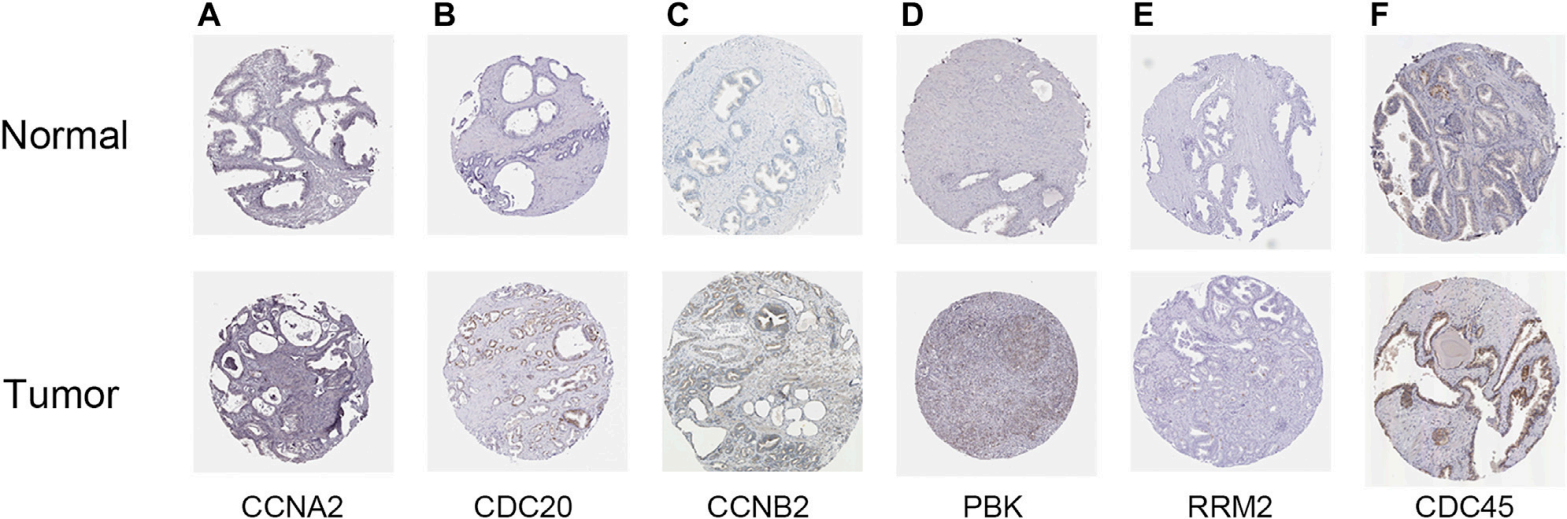
Validation of the expression of hub genes available in prostate cancer (PCa) and normal tissues in the Human Protein Atlas (HPA) database. **(A)** CCNA2, **(B)** CDC20, **(C)** CCNB2, **(D)** PBK, **(E)** RRM2, and **(F)** CDC45.

To further verify the relationship between these eight hub genes and PCa occurrence and progress, the mRNA expression of the eight hub genes correlated with sample types and clinical characters based on the TCGA-PRAD dataset were analyzed. *BUB1*, *KIF2C*, *CDC20*, and *PBK* were significantly highly expressed in tumor tissues, the older age group, and advanced stages of PCa (*p* < 0.05, Figure 9), which is in accordance with the results displayed in Figure 2, indicating that *BUB1*, *KIF2C*, *CDC20*, and *PBK* might promote the occurrence and progress of PCa. Subsequently, we investigated the protein expression of hub genes (BUB1 and KIF2C were unavailable) based on the immunohistochemistry results downloaded from the HPA database. All six hub genes were upregulated in PCa tissues (Figure 10). These results revealed that the expression of these genes was indeed upregulated at both transcript and translation levels in PCa. The eight hub genes were the candidate biomarkers for PCa.

### RRM2 Might Be a Potential Biomarker for PCa Prognosis

**FIGURE 11:**
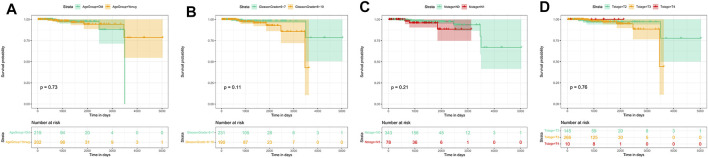
Survival analysis by clinical data stratification of prostate cancer. **(A)** Age of PCa patients (cutoff: 62). **(B)** Gleason stage of tumor. **(C)** N stage of tumor. **(D)** T stage of tumor.

**FIGURE 12:**
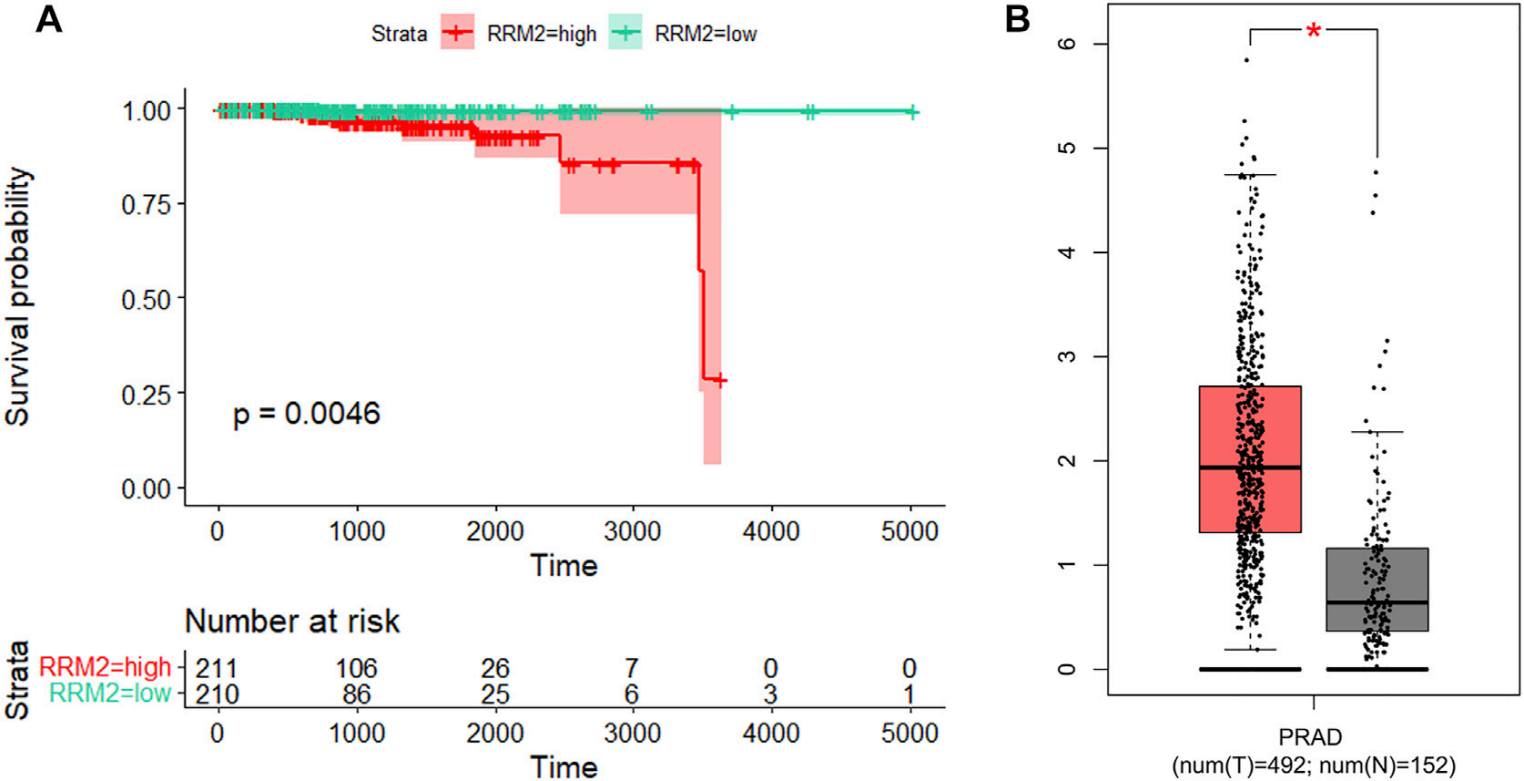
Survival analysis and expression analysis of *RRM2*. **(A)** Association between *RRM2* expression and survival outcomes of TCGA-PRAD cohort. **(B)** mRNA expression levels of *RRM2* in the GEPIA database.

Prognosis is an important aspect in cancer treatment. Unfortunately, the survival analysis of age, Gleason stage, T stage, and N stage of PCa patients showed no statistical significance with the overall survival of prostate cancer patients (*p* > 0.05, Figure 11), indicating that it may be difficult to predict the prognosis of prostate cancer patients merely based on clinical manifestations. For the eight hub genes, a survival analysis was performed, which provided a value of a cut point that corresponds to the most significant relation with the outcome. The KM curve with the risk table is shown in Figure 12. We found that *RRM2* was statistically significant to the prognosis of prostate cancer patients (*p* < 0.05) (Figure 12A). The expression of *RRM2* between normal tissues and tumor tissues was also validated in the GEPIA database (Figure 12B). All results revealed that the high expression of *RRM2* led to poor prognosis, indicating that *RRM2* may be an independent potential biomarker of PCa prognosis.

## Discussion

The prediction for the prognosis of PCa patients is relatively difficult compared with other tumors. Consequently, it is urgent to detect biomarkers for the early diagnosis and treatment of prostate cancer. Through the KEGG pathway analysis, we found that the pathways associated with DEGs in the key module are cell cycle, oocyte meiosis, progesterone-mediated oocyte maturation, and the p53 signaling pathway. Oocyte meiosis and progesterone-mediated oocyte maturation were reported to be related with reproductive inheritance, which were widely studied in women (Jaffe and Egbert, 2017; Lin T. et al., 2019). However, our study revealed that the genes associated with PCa were numerously enriched in these two pathways, indicating that there may be potential new features related with men, and further research was expected to be performed for more explorations. Furthermore, numerous studies with reliable experiments have demonstrated that the activation of the p53 signaling pathway is correlated with the development of various types of cancer, including prostate cancer (Gao et al., 2014; Gao et al., 2020; Zheng et al., 2020; Dong et al., 2021). In addition, p53 has been identified to be associated with cell cycle and apoptosis, and activation of the p53 tumor suppressor can lead to cell cycle arrest (Wang et al., 2015; Engeland, 2018). In our study, as the network relationship of KEGG pathway enrichment analysis showed, *CDK1*, *RRM2*, *CCNB2*, and *GTSE1* were screened out to be associated with the p53 signaling pathway. Furthermore, *GTSE1* has been experimentally validated to play an important role in the p53 signaling pathway. Lin et al. revealed that *GTSE1* could regulate the p53 function to alter the cell cycle distribution dependent on the mutation state of p53 in breast cancer (Lin F. et al., 2019). *CDK1*, *RRM2*, and *CCNB2* were reported to be correlated with the p53 signaling pathway, which may be urgent to be validated with reliable experiments for further analysis (Fischer et al., 2016; Yang et al., 2019; Jin et al., 2020). Furthermore, the network relationship of GO enrichment analysis was screened to visualize enrichment results, which may provide more possibility for the research of key genes in prostate cancer.

Through the application of Cox proportional hazard regression analysis, we identified eight genes for the prognostic prediction of prostate cancer, including *BUB1*, *KIF2C*, *CCNA2*, *CDC20*, *CCNB2*, *PBK*, *RRM2*, and *CDC45*. The accuracy of our model based on these genes was relatively high. The results of the KM curves (*p* < 0.05), C-index (0.82), AUC of the train set (0.825), and AUC of the test set (0.795) revealed that the signature may count for categorizing patients into high-risk and low-risk groups and act as an effective indicator of prognosis. Based on WGCNA module selecting and gene expression analysis, *BUB1*, *KIF2C*, *CDC20*, and *PBK* were eventually detected to be potentially associated with the clinical pathological features of prostate cancer. However, experimental validation of BUB1 and KIF2C in prostate cancer was unavailable in the HPA database, which were valuable to be verified based on more experiments.

In our study, *BUB1*, *CDC20*, *PBK*, and *CDC45* were considered as potential tumor suppressor genes. Budding uninhibited by benzimidazoles 1 (BUB1), a mitotic checkpoint serine/threonine kinase, has been reported in numerous cancer studies. *BUB1* was highly expressed in gastric cancer, and the overall survival time was prolonged in gastric cancer patients with a high expression of *BUB1* (Li X. et al., 2020). However, it was verified that the high expression of BUB1 was correlated with poor prognosis in hepatocellular carcinoma (HCC) (Yang et al., 2019). In contrast, *BUB1* was considered a protective prognosis gene in our study and enriched in the p53 signaling pathway, indicating that the complexity of the gene requires a lot of validation. Also, the mechanism of BUB1 in regulating the development of prostate cancer remains to be explored. Cell division cycle 20 (CDC20) is an anaphase-promoting complex activator that plays a key role in cell division and tumorigenesis. Recent studies indicated that *CDC20* may serve an oncogenic role in various types of human cancer. *CDC20* has been reported to serve as an independent predictor for biochemical recurrence (BCR) in prostate cancer (Li et al., 2016; Mao et al., 2016). Furthermore, it was reported that *CDC20* overexpression facilitates the docetaxel resistance of the castration-resistant prostate cancer (CRPC) cell lines in a Bim-dependent manner, indicating that the drugs targeting *CDC20* were urgent to be developed for the treatment of the CRPC with docetaxel resistance (Wu et al., 2018). PDZ binding kinase (PBK) is a serine/threonine kinase. *PBK* was reported to regulate the expression of androgen receptor (AR) protein, and the overexpression of PBK in aggressive prostate cancer was reported to be associated with early biochemical relapse and poor clinical manifestations (Warren et al., 2019). However, the mechanism has not been revealed. Moreover, PBK was a downstream target of *RORγ* that exerted the cellular effects, indicating that *PBK*, *RORγ*, and *AR* were all associated with the growth and survival of aggressive prostate cancer (Zhang et al., 2021). However, possible mechanisms in non-aggressive prostate cancer have not been researched. Cell division cycle 45 (CDC45) plays a critical role in DNA replication. *CDC45* was reported to be a potential prognostic and diagnostic biomarker in colorectal cancer (CRC) and HCC, but few mechanisms have been found (Hu et al., 2019; Lu et al., 2021). In addition, *CDC45* was only detected to be potentially correlated with prostate cancer based on co-expression network analysis and functional enrichment analysis, indicating that more empirical evidence was expected to validate the functions of *CDC45* in prostate cancer (Cai J. et al., 2020; Wei J. et al., 2020; Wang and Yang, 2020).

Furthermore , *KIF2C*, *CCNA2*, *CCNB2*, and *RRM2* were found to be potential risk genes in our study. Kinesin Family Member 2C (KIF2C) is a modulator in microtubule depolymerization, bipolar spindle formation, and chromosome segregation. It has been reported to be associated with the prognosis of numerous cancers. It was reported that *KIF2C* expression was significantly upregulated in HCC and breast cancer, and that *KIF2C* up-regulation was associated with a poor prognosis (Wei S. et al., 2020; Li T. et al., 2020). However, there was also evidence that *MCAK*/*KIF2C* played an important role in the regulation of cellular senescence through a p53-dependent pathway and might contribute to tissue/organism aging and protection of cellular transformation (Gwon et al., 2012). In our study, *KIF2C* performed as a risk prognostic factor, but no prognostic value of *KIF2C* in prostate cancer has been verified. Cyclin A2 (CCNA2) belongs to the highly conserved cyclin family and is significantly overexpressed in various cancer types. *CCNA2* mRNA was reported to be distinctly upregulated in non-small-cell lung cancer (NSCLC) specimens and cell lines, but no prognostic value of *CCNA2* in NSCLC has been found (Liu et al., 2019; Du et al., 2020). In our study, *CCNCA2* was associated with the prognosis of prostate cancer and was statistically significant in Cox regression analysis (*p* < 0.05). Our result has been validated from the research conducted by Yang et al. (2020), indicating that *CCNA2* is probably a key gene of prostate cancer. Cyclin B2 (CCNB2) performs as a member of cyclin family proteins, serving a key role in the progression of G2/M transition. It was proved that the knockdown of circ_*CCNB2* increased the radiosensitivity of PCa through repressing autophagy by the miR-30b-5p/KIF18A axis, but no biological function of *CCNB2* in prostate cancer has been found (Cai F. et al., 2020). *CCNB2* was highly expressed in human triple-negative breast cancer (TNBC) tissues and correlated with the prognosis and clinical pathological features (Wu et al., 2021). However, the key targets of *CCNB2* in TNBC and its biological functions remain to be found. Ribonucleotide reductase regulatory subunit M2 (RRM2) is a rate-limiting enzyme involved in DNA repair and synthesis. *RRM2* has been reported in various types of cancer and has been implicated in tumor progression. In lung adenocarcinoma (LUAD), the overexpression of *RRM2* was an independent predictive factor of poor prognosis, which increased the activation of Bcl-2 and E-cadherin signaling pathways and reduced the activation of the p53 signaling pathway (Jin et al., 2020). In prostate cancer, it has been reported that the increased expression of *RRM2* was associated with poor prognosis, which was also validated in our study (Mazzu et al., 2019). In addition, we found that *RRM2* was enriched in the p53 signaling pathway, indicating that the mechanism of *RRM2* in the development of prostate cancer was potentially correlated with the p53 signaling pathway, which remained unclear due to lack of experimental validation.

## Conclusion

In summary, we found a key DEG module associated with prostate cancer. Then, the network relationship of GO enrichment and KEGG enrichment was screened. Next, we found eight hub genes and constructed a risk model to predict the prognosis of prostate cancer, providing a clue for risk stratification and prognosis prediction in prostate cancer. Finally, *RRM2* was enriched in the p53 signal pathway and its expression was statistically significant to the prognosis of prostate cancer, indicating that *RRM2* may play a key role in the progression of PCa. Nevertheless, our research was based on bioinformatic analysis, and further experimental exploration is needed to illustrate the mechanism of *RRM2* in PCa.

## Data Availability

The original contributions presented in the study are included in the article/Supplementary Material, further inquiries can be directed to the corresponding authors.
